# Prognosis prediction for end-stage pancreatic cancer with home medical care: A retrospective cohort study conducted at multiple institutions in Hiroshima

**DOI:** 10.1186/s12904-026-02015-1

**Published:** 2026-05-11

**Authors:** Hiroaki Yamane, Aki Yoshimitsu, Hiroshi Ota, Etsushi Akimoto, Tomoyuki Abe, Jun Kawamoto, Ryo Sakai, Akihiko Yamashina, Yoshiko Mikami, Takeshi Yamaguchi, Yasutaka Oda, Motoi Yamane, Noriyoshi Maruyama

**Affiliations:** 1 Yamane clinic, Hiroshima, Japan; 2 Department of Clinical Oncology, JA Hiroshima General Hospital, Hiroshima, Japan; 3 Akimoto clinic, Hiroshima, Japan; 4 Department of Gastroenterological and Transplant Surgery, Hiroshima University Hospital, Hiroshima, Japan; 5 Amano Rehabilitation Hospital, Hiroshima, Japan; 6 Akashi Medical clinic, Hiroshima, Japan; 7 Home care clinic Momiji, Hiroshima, Japan; 8 Mikami home clinic, Hiroshima, Japan; 9 Yamaguchi home care clinic, Hiroshima, Japan; 10 Call Medical Clinic Hiroshima, Hiroshima, Japan; 11 Maruyama home clinic, Hiroshima, Japan

**Keywords:** End-stage cancer, Home medical care, Palliative care at home, Prognosis, Pancreatic cancer

## Abstract

**Background:**

Home medical care provision has become more significant as the Japanese population continues to age. However, the number of patients with end-stage pancreatic cancer receiving home medical care remains limited, regardless of the patients’ strong preference for home medical care. Additionally, prognostic prediction in patients with end-stage pancreatic cancer receiving home medical care is not well validated, which limits the ability of clinicians to plan effective palliative interventions. Therefore, in this study, we aimed to identify the prognostic factors associated with overall survival (OS) in patients with end-stage pancreatic cancer receiving home medical care and to develop a new prognostic scoring system.

**Methods:**

This nine-center, retrospective study included patients with end-stage pancreatic cancer who were treated with home medical care between 2021 and 2025 in Japan. Univariate and multivariate analyses were performed to identify independent prognostic factors associated with OS. A prognostic scoring system was developed based on the factors identified in the multivariate analysis.

**Results:**

In total, 168 (76 male and 92 female) participants were included in this study. The median OS with home medical care was 30 days. Multivariate analysis revealed that a history of opioid use before the initiation of home medical care, the presence of peritoneal dissemination, and a modified Glasgow Prognostic Score (mGPS) of 2 were independently associated with poor OS at home. Stratification based on the above factors demonstrated that patients presenting with two or more risk factors had significantly worse home survival than did those with one or no risk factors (24 versus 45 days, *p* < 0.001).

**Conclusions:**

This study identified prior opioid use, peritoneal dissemination, and a mGPS of 2 as independent risk factors for OS in patients with end-stage pancreatic cancer receiving home medical care. These results indicate that assessing these factors may aid healthcare providers in making informed decisions and optimizing supportive care strategies tailored to patient prognosis in the home medical care setting.

**Supplementary Information:**

The online version contains supplementary material available at 10.1186/s12904-026-02015-1.

## Background

Home medical care in Japan is important in clinical settings because of an increasing older population. Notably, home medical care costs less than hospitalization and results in symptom improvement; thus, it is expected to become a main choice of treatment for older adults in the future [1]. However, patients with a short life expectancy may experience increased anxiety about end-of-life care at home owing to their inability to establish sufficient trust with healthcare professionals [2]. Additionally, survival at home for patients with end-stage cancer would be worse than that of patients with other diseases [3]. Therefore, clinicians should precisely predict the prognosis of patients with end-stage disease to facilitate an early transition to home medical care, allowing time to plan for appropriate care, prepare the home environment, and support the emotional needs of patients and caregivers. However, only a few reports are available on this topic, resulting in insufficient evidence [4].

Pancreatic cancer is a leading cause of cancer-related death worldwide, and its global burden has more than doubled over the past 25 years [5]. This is because despite advancements in multidisciplinary treatments such as chemotherapy and surgery, pancreatic cancer has a poor malignant potential [5]. Inflammatory-related predictive scores, such as C-reactive protein (CRP) and serum albumin (Alb), which evaluate the nutritional status, as well as combinations of these factors, have been identified as prognostic predictors in many cancers [6–8]. However, most of these predictive scoring systems have been applied to evaluate prognosis after surgery or the effectiveness of chemotherapy [6, 8], and their utility in the context of home care, particularly in patients with end-stage pancreatic cancer, remains unknown.

Therefore, we hypothesized that specific clinical and biological parameters available at the time of transition to home medical care could predict overall survival (OS) in patients with end-stage pancreatic cancer. Hence, in this study, we aimed to identify independent prognostic factors for patients with end-stage pancreatic cancer transitioning to home medical care and develop a simple, clinically applicable scoring system to stratify patients according to their expected survival.

## Methods

### Patients

This multi-center retrospective study enrolled patients with end-stage pancreatic cancer from 9 centers in Japan who were treated at home between January 2021 and April 2025. The inclusion criteria were as follows: diagnosis of end-stage pancreatic cancer, age > 18 years, and being cared for at home or in Serviced Housing for Older People [9]. In this study, end-stage pancreatic cancer was defined as disease in which patients were no longer candidates for disease-modifying treatments owing to rapid clinical deterioration, poor performance status (PS) (Eastern Cooperative Oncology Group PS of 3–4), refractory symptoms, or exhaustion of available chemotherapy options, as determined by the attending oncologist. Additionally, cases where treatment was discontinued at the patient’s request and home medical care was desired were also defined as terminal. Disease staging was performed in accordance with the guidelines of the 8th Union for International Cancer Control guidelines [10]. Pancreatic ductal adenocarcinoma was defined as pancreatic cancer. Patients transferred to palliative care units or hospitals were excluded from the study.

The need for individual participant consent was waived using the opt-out method. This study was approved by the Medical Governance Research Institute (approval number: MG2024-02-R2). All methods were performed in accordance with the relevant guidelines and regulations.

### Treatment

During home medical care, the treatment focuses on palliative care along with management to alleviate symptoms. Therefore, patients were treated based on their cancer-related symptoms, including the administration of strong opioids and initiation of home oxygen therapy (HOT) as required. Chemotherapy at home was administered upon the referring physician’s discretion. In Japan, clinicians are encouraged to attend educational lectures to gain foundational knowledge on opioid use.

### Data collection

At the beginning of home medical care, we collected data on the patients’ background factors, such as age, sex, place of death, PS, whether the patient had undergone pancreatic resection for pancreatic cancer, and location of metastasis. In addition, we collected information on edema (trunk or limbs), delirium, dyspnea at rest, and oral intake. Oral intake was classified into normal intake to moderate reduction (more than a few bites) and severe reduction (a few bites) [11]. Other factors related to the home environment and palliative treatment, such as the presence of primary caregivers, family relationships, whether the patients lived with family/partner or alone, and usage of strong opioids and HOT, were also collected. Data on tumor characteristics, such as Tumor, Node, Metastasis classification [10], primary tumor location, histological type, and distant metastasis, were also collected. Blood examination data were obtained from the referring institution approximately 1 week prior to the start of home medical care or through examinations conducted at the home support clinic. Additionally, the modified Glasgow Prognostic Score (mGPS) was calculated. The mGPS ranges from 0 to 2, with GPS0 defined as Alb ≥ 3.5 g/dL and serum CRP < 0.5 mg/dL, GPS1 as serum Alb < 3.5 mg/dL or serum CRP ≥ 0.5 mg/dL, and GPS2 as serum Alb < 3.5 mg/dL and serum CRP ≥ 0.5 mg/dL [7]. The neutrophil-to-lymphocyte ratio (NLR) was calculated by dividing the absolute neutrophil count by the absolute lymphocyte count [6]. OS was defined as the period from the start of home medical care until the date of death. For transparency, the number of missing values for each variable is presented in Tables 1 and 2. Missing values were excluded from the analysis.

**Table 1:** Background information on patients with end-stage pancreatic cancer

Variable	Detail of variable	*N* (% or interquartile range)
Age	Median age, y	75 (68–82)
	> 75 y /≤ 75 y	82 (48.8%)/86 (51.2%)
Sex	Male/female	76 (45.2%)/92 (54.8%)
Comorbidity	Hypertension	55 (32.7%)
	Diabetes mellitus	59 (35.1%)
	Dementia	17 (10.2%)
Location of death	Home	165 (98.2%)
	Serviced Housing for Older People	3 (1.8%)
Overall survival at home	Median, d	30 (15–53)
Condition at time of visit	PS 0/1/2/3/4	5 (3.0%)/24 (14.3%)/55 (32.7%)/57 (33.9%)/27 (16.1%)
	Oral intake: moderate reduction/severe reduction	110 (65.5%)/58 (34.5%)
	Delirium	25 (14.9%)
	Dyspnea at rest	25 (14.9%)
	Cancer-related pain	145 (86.3%)
Primary caregiver	Spouse	99 (58.9%)
	Child	64 (38.1%)
	Brother	3 (1.8%)
	Other	2 (1.2%)
Cohabitation with primary caregivers	Yes	146 (86.9%)
Cancer treatment during home medical care	Yes	3 (1.8%)
Palliative care intervention before home medical care	Yes	76 (45.2%)
Sedation	Yes	85 (50.6%)
Strong opioid usage	Yes	146 (86.9%)
Time of opioid initiation	Before home medical care	83 (56.8%)
Opioid CSI	Yes	60 (35.7%)
HOT	Yes	35 (20.8%)
Time of HOT initiation	Before home medical care	8 (4.8%)
	After home medical care	27 (16.1%)

*Abbreviations*: *CSI* Continuous subcutaneous injection, *HOT* Home oxygen therapy, *PS* Performance status

**Table 2:** Tumor-related and blood examination data of patients with end-stage pancreatic cancer

Variable	Median (IQR)	Number of NA (%)
Primary TNM stage	1/2/3/4/NA	3 (1.8%)/26 (15.5%)/12 (7.1%)/93 (55.4%)/34 (20.2%)
Location of primary tumor	Head/body/tail/NA	66 (39.3%)/32 (19.0%)/29 (17.3%)/41 (24.4%)
History of pancreatectomy	Yes	41 (24.4%)
Postoperative recurrence	Yes/NA	30 (17.9%)/9 (5.4%)
Number of metastases	Median number	1 (1–2)
Site of metastasis	Liver	97 (57.7%)
	Peritoneum	57 (33.9%)
	Lymph nodes	47 (28.0%)
	Lungs	37 (22.0%)

Variable	Median (IQR)	NA
WBC count (/µL)	7505 (5622–10955)	0 (0%)
Albumin (g/dL)	2.8 (2.2–3.4)	0 (0%)
CRP (mg/dL)	2.3 (0.9–6.1)	0 (0%)
NLR	5.1 (3.3–9.0)	0 (0%)
mGPS 0/1/2	12 (7.1%)/35 (20.8%)/121 (72.0%)	0 (0%)
CEA (ng/mL)	10.2 (3.2–48.3)	95 (56.5%)
CA19-9 (U/mL)	2505 (63–8621)	102 (60.7%)

*Abbreviations*: *CA19-9* Carbohydrate antigen 19 − 9, *CEA* Carcinoembryonic antigen, *CRP* C-reactive protein, *IQR* Interquartile range, *NA* Not available, *NLR* Neutrophil-to-lymphocyte ratio, *WBC* White blood cell

### Evaluation and statistical analysis

Continuous variables were expressed as median values with interquartile ranges (IQRs). The variables were divided into two groups based on their median values, and survival curves were constructed using the Kaplan–Meier method. The two groups were evaluated using the generalized Wilcoxon test, wherein survival time was considered the dependent variable for each observation. Cox univariate regression analysis was performed to identify the prognostic factors for survival in the study population. Subsequently, multivariate analysis was performed by incorporating significant variables from the univariate analysis. Hazard ratios (HRs) and 95% confidence intervals (CIs) were also calculated. Statistical significance was set at *p* = 0.050. To assess the robustness of the prognostic association of the NLR, additional sensitivity analyses were performed using (1) a clinically reported cutoff of NLR > 5 [12] and (2) a continuous specification modeled as a log2-transformed NLR (interpreted per doubling). All covariates included in the primary multivariable model were retained. All statistical analyses were performed using JMP version 15 (SAS Institute, Cary, NC, USA).

## Results

### Patient background at the beginning of home medical care

In this study, data were collected from 190 patients. After removing all patients with missing data, analysis was performed on the data from 168 patients. Table 1 presents the characteristics of the 168 consecutive patients (median age, 75 years; IQR: 68–82 years) enrolled in this study. Among them, 76 were male (45.2%) and 92 were female (54.8%). Comorbidities included hypertension in 55 patients (32.7%) and dementia in 17 patients (10.2%). The median OS after initiating home medical care was 30 days (IQR: 15–53 days). Among the 168 patients, 54 had severely reduced oral intake (34.2%) and 145 (86.3%) experienced cancer-related pain.

Of the primary caregivers, 58.9% were spouses and 38.1% were sons and daughters. Only 3 patients continued anticancer drug therapy at home (1.8%). Strong opioids were administered to 146 patients (86.9%), with 83 patients (56.8%) having started opioid use before receiving home medical care. HOT was administered to 35 patients (20.8%). The most common site of distant metastasis was the liver (97 patients, 57.7%), followed by the peritoneum (57 patients, 33.9%), lymph nodes (47 patients, 28%), and lungs (37 patients, 22%). The blood examination data are summarized in Table 2. The median NLR was 5.1 (3.3–9.0). Overall, 12 patients (7.1%) had an mGPS of 0, 35 (20.8%) had a score of 1, and 121 (72.0%) had a score of 2.

### Univariate analysis of prognostic evaluation for OS with home medical care

The results of the univariate analysis comparing OS and other variables are shown in Table 3. The younger group (aged < 75 years) had a shorter OS than the older group did (aged ≥ 75 years) (*p* = 0.002). Patients with a PS of 4 and severely impaired oral intake experienced poor outcomes. Patients with cancer-related pain had worse OS than did those without pain (*p* = 0.017). However, no significant correlation was found between delirium and respiratory distress at rest in home-based OS. Additionally, no significant differences were observed in OS at home based on the type of primary caregiver or cohabitation status. Patients with liver metastasis (*p* < 0.001) or peritoneal dissemination (*p* < 0.001) had a poor prognosis. Although a history of strong opioid treatment did not affect OS, patients who used opioids prior to receiving home treatment had worse OS (*p* = 0.002) than did those who received opioids only during treatment. Additionally, blood examination data showed that an NLR > 5.1 (*p* < 0.001) and an mGPS of 2 (*p* < 0.001) affected OS.

**Table 3:** Univariate analysis of sociodemographic and clinical variables, clinical symptoms, and tumor factors

Variable	Median OS (IQR) (d)	*p* value
**Age: > 75 y/≤ 75 y**	**38 (22–68) / 26 (13–39)**	**0.002**
Sex: male/female	34 (17–63.5)/27 (14.5–44)	0.084
Hypertension: yes/no	**37 (22–70)/27 (14–48)**	**0.013**
Diabetes mellitus: yes/no	32 (15–56)/27 (16–49)	0.395
Dementia: yes/no	30 (15–91)/31 (16–52)	0.508
PS: 4/≤ 3	**22 (16–42)/32 (18–53)**	**0.011**
Oral intake: severe/moderate	**20 (11–37)/35 (22–67)**	**< 0.001**
Delirium: yes/no	27 (11–44)/31 (16–54)	0.523
Dyspnea at rest: yes/no	25 (16–37)/32 (15–56)	0.160
Cancer related-pain: yes/no	**29 (15–49)/51 (23–111)**	**0.017**
Primary caregiver: spouse/child/brother/other	30 (18–52)/31 (11–65)/19 (15–333)/26 (14–38)	0.966
Cohabitation with primary caregiver: yes/no	32 (16–53)/26 (11–49)	0.598
Palliative care intervention before home medical care: yes/no	32 (17–52)/30 (15–54)	0.752
Sedation: yes/no	32 (16–53)/30 (15–53)	0.917
Usage of strong opioid: yes/no	30 (16–52)/32 (15–79)	0.392
Use of opioid before home medical care: yes/no	**25 (13–38)/38 (19–70)**	**0.002**
CSI: yes/no	27 (15–43)/35 (17–62)	0.218
HOT: yes/no	26 (16–52)/32 (15–53)	0.496
History of pancreatectomy: yes/no	27 (12–38)/32 (15–53)	0.505
Distant metastasis: yes/no	29 (15–50)/37 (17–90)	0.083
Liver metastasis: yes/no	**27 (15–39)/38 (19–85)**	**0.005**
Peritoneal dissemination: yes/no	**25 (11–37)/35 (19–65)**	**0.002**
Lymphatic metastasis: yes/no	27 (15–49)/32 (16–61)	0.356
Lung metastasis: yes/no	29 (21–45)/32 (14–56)	0.891
NLR: > 5.1/≤ 5.1	**23 (11–38)/39 (24–82)**	**< 0.001**
mGPS: 2/0 & 1	**26 (13–38)/63 (32–116)**	**< 0.001**

Bold text indicates statistical significance

*Abbreviations*: *PS* Performance status, *HOT* Home oxygen therapy, *CSI* Continuous subcutaneous injection, *NLR* Neutrophil-to-lymphocyte ratio, *mGPS* Modified Glasgow Prognostic Score, *IQR* Interquartile range, *OS* Overall survival

### Multivariate analysis of OS and score of predictive prognoses with home medical care

**Fig. 1:**
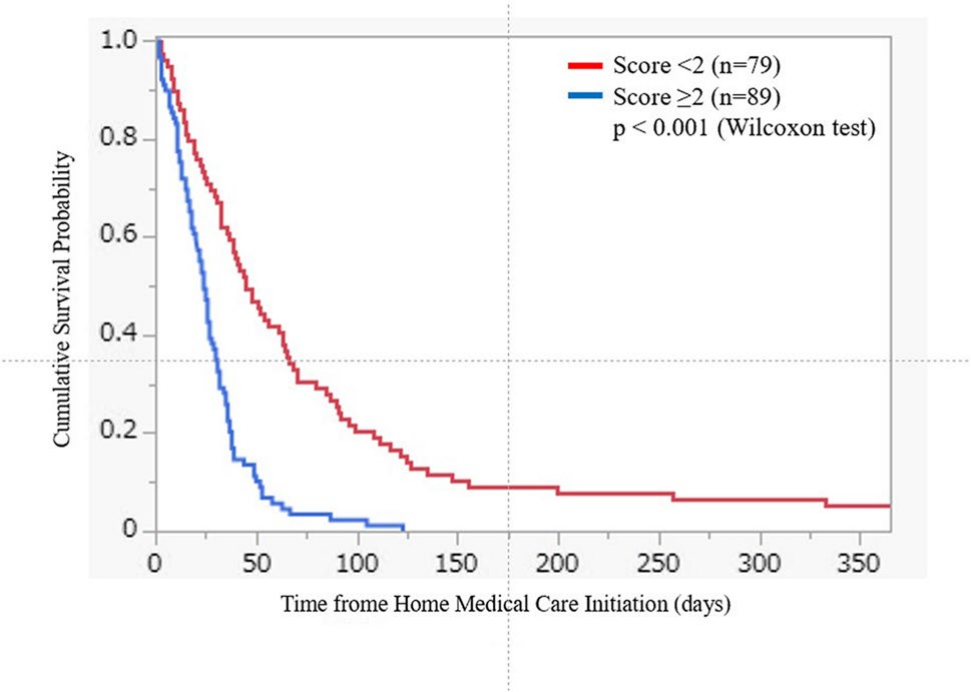
Kaplan–Meier survival curves based on the prognostic score. Kaplan–Meier survival curves of patients with end-stage pancreatic cancer receiving home medical care stratified by prognostic scores. Patients with a score ≥ 2 (blue line, *n* = 89) had significantly shorter survival than did those with a score < 2 (red line, *n* = 79) (*p* < 0.001, generalized Wilcoxon test). Median OS: 45 days for the low-risk group (score < 2) vs. 24 days for the high-risk group (score ≥ 2)

The results of the multivariate analysis are presented in Table 4. The analysis identified three independent variables associated with OS: use of opioids before home medical care (HR: 1.44, *p* = 0.047), peritoneal dissemination (HR: 1.50, *p* = 0.020), and an mGPS of 2 (HR: 2.16, *p* < 0.001). Subsequently, each factor was assigned a score of 1 point, with the total score ranging from 0 to 3 points. Patients were then stratified based on the total score as follows: 0 points, *n* = 20 (11.9%); 1 point, *n* = 59 (35.1%); 2 points, *n* = 65 (38.7%); and 3 points, *n* = 24 (14.3%). Finally, patients were grouped into low-risk (0–1 point, *n* = 79) and high-risk (2–3 points, *n* = 89) cohorts. The median OS for low-risk and high-risk patients was 45 days (IQR: 22–91 days) and 24 days (IQR: 13–36 days), respectively (*p* < 0.001) (Fig. 1).

**Table 4:** Multivariate analysis of factors affecting home survival duration

Variable	Hazard ratio	95% confidence interval	*p* value
Age > 75 y	0.923	0.64–1.33	0.668
Hypertension	0.821	0.57–1.18	0.285
PS of 4	1.35	0.95–1.92	0.180
Oral intake, severe reduction	1.35	0.95–1.92	0.098
Cancer-related pain	1.387	0.82–2.35	0.224
**Use** **of opioids before home medical care**	**1.44**	**1.00–2.07**	**0.047**
Liver metastasis	1.36	0.95–1.94	0.088
**Peritoneal dissemination**	**1.50**	**1.07–2.11**	**0.020**
NLR > 5.1	1.33	0.93–1.91	0.118
**mGPS of 2**	**2.16**	**1.43–3.28**	**< 0.001**

Bold text indicates statistical significance

*Abbreviations*: *PS* Performance status, *NLR* Neutrophil-to-lymphocyte ratio, *mGPS* Modified Glasgow Prognostic Score

### Sensitivity analyses of the NLR

In sensitivity analyses, the prognostic effect of the NLR remained directionally consistent across all model specifications. When the NLR was dichotomized using a clinically reported threshold of > 5, it was found to be significantly associated with shorter survival (HR: 1.52, 95% CI: 1.05–2.20). When modeled as a continuous variable using a log2-transformed NLR, the association was also significant (HR: 1.22 per doubling, 95% CI: 1.09–1.36) and demonstrated the best overall model fit (Akaike information criterion = 1332.6). Differences in statistical significance observed in the median-based model were attributable to information loss inherent to dichotomization rather than to instability of the prognostic association. The results of these sensitivity analyses are summarized in Additional File 1, Supplementary Table S1.

## Discussion

In this study, we examined real-world data of patients with end-stage pancreatic cancer who received palliative home medical care. Our results identified use of opioids before home medical care, peritoneal dissemination, and an mGPS of 2 as independent poor prognostic factors for OS in this patient population. Subsequently, we developed a prognostic scoring system based on these factors that effectively stratified patients into low-risk and high-risk cohorts, demonstrating significantly different median OS. Thus, our results suggest that this simple, clinically applicable scoring system would assist clinicians in anticipating patient trajectories and optimizing supportive care strategies in the home medical care setting.

The multivariate analysis results identified that an mGPS of 2 is an independent prognostic factor for dismal prognosis. For instance, inflammatory-related prognostic scores, including the mGPS are recognized predictors in various cancers, often evaluating prognosis after surgery or chemotherapy effectiveness [6, 8]. However, the utility of the mGPS specifically in the context of home medical care for patients with pancreatic cancer was previously unclear. In this regard, our study provides novel evidence supporting its value in this specific population in the context of home medical care. Additionally, our study indicates that an unfavorable prognosis is associated with opioid use before home medical care. Several studies have demonstrated that a history of opioid use does not affect prognosis [13]. This finding contradicts our results; however, no previous report has exclusively focused on terminally ill patients with cancer receiving home-based care. Additionally, the association between prior opioid use and poor prognosis may reflect more aggressive tumor biology or greater symptom burden before the transition to home care rather than a direct prognostic effect of opioids. However, we could not adjust for opioid dosage, pain intensity, or symptom severity, which may have confounded this relationship. Further prospective studies are needed regarding opioid use history.

Moreover, peritoneal dissemination was recognized as a negative prognostic factor in patients with pancreatic cancer receiving home medical care. This finding is consistent with the fact that peritoneal dissemination indicates advanced disease and is associated with tumor aggressiveness, often leading to debilitating complications, such as ascites or cachexia [14]. The inclusion of this factor in our prognostic score provides a valuable indicator for tailoring intensive palliative interventions in the home setting.

The prognostic score developed in this study is based on three readily available factors and may help clinicians, caregivers, and patients align expectations at the start of home medical care. By identifying patients with poorer expected survival (e.g., those scoring ≥ 2 points), clinicians can consider earlier referrals to hospice services, prioritize family discussions, and make logistical arrangements without delay. Home doctors should be encouraged to increase the frequency of home visits for patients with scores of two or higher. This proactive approach would help establish trust with patients and their families early on. In contrast, patients with lower scores could benefit from a more sustained palliative care plan focused on maintaining function and comfort. Although our score showed potential for stratifying patients by expected survival, its clinical utility should be interpreted cautiously given this study’s retrospective design and lack of validation. Prospective studies are required before the model can be widely applied.

Interestingly, younger patients (aged < 75 years) exhibited shorter OS, compared with older patients (aged > 75 years). Additional analysis revealed that the younger patient group included a higher proportion of patients with cancer-related pain and more patients for whom opioid use had been initiated prior to home medical care, compared with the older patient group (Additional File 1, Supplementary Table S2). The blood examination results of younger patients were also worse compared with those of older patients. Distant metastasis was also more common in the younger patient group than in the older patient group. One possible explanation is that younger patients may have received more aggressive anticancer treatments before home transition, resulting in more advanced disease at the time of referral. Alternatively, the referral patterns may have differed, with older patients being referred earlier for supportive home care. However, the reviewed patient data lacked information on the content and duration of treatment history and cancer stage, making these areas subjects for future prospective research.

This study has some limitations. First, its retrospective design may have led to incomplete documentation and unmeasured confounding, including lack of detailed treatment history, tumor markers, nutritional indicator and timing from diagnosis. The association between the NLR and survival was generally robust to alternative model specifications. Although median dichotomization yielded a weaker association, both the clinically reported cutoff of NLR ≥ 5 and log2-transformed continuous model demonstrated significant prognostic value, with comparable or better model fit. These findings indicate that the impact of the NLR is not dependent on the particular choice of cutoff and that modest variability in significance largely reflects information loss caused by dichotomization. Modeling the NLR as a continuous variable (per doubling) provided the most stable and clinically interpretable estimate. Several clinical variables used in our score, such as oral intake, delirium, edema, and dyspnea, are inherently subjective and may involve inter-observer variability. However, these assessments follow standardized definitions routinely used in home-based palliative practice and are unlikely to have substantially influenced the observed prognostic associations. Second, the sample size was modest, and all participating centers were located within Hiroshima, which may limit generalizability. Third, although all participating centers belonged to the same regional home-care network and followed standardized clinical protocols, residual heterogeneity in practice patterns may still have influenced the findings. Finally, although we identified three independent prognostic factors, the scoring system lacks internal and external validation and may be overfitted to this cohort. Prospective studies with larger, diverse populations are needed to confirm its clinical utility.

## Conclusions

Our findings revealed that a history of opioid use before home medical care, peritoneal dissemination, and an mGPS of 2 are valuable prognostic markers for predicting outcomes in patients with end-stage pancreatic cancer receiving home medical care. Patients with these factors can be expected to have shorter OS, making our prognostic model highly significant for determining treatment strategies in home medical care. 

## Supplementary Information

Additional file 1: Supplementary Table S1. Sensitivity analyses comparing model specifications. Supplementary Table S2. Comparison with patients aged ≥ 75 years.

## Data Availability

The datasets generated and/or analyzed during the current study are available from the corresponding author on reasonable request.

## Abbreviations

Alb: Serum albumin
CI: Confidence interval
CRP: C-reactive protein
HOT: Home oxygen therapy
HR: Hazard ratio
IQR: Interquartile range
mGPS: Modified Glasgow Prognostic Score
NLR: Neutrophil-to-lymphocyte ratio
OS: Overall survival
PS: Performance status
