# Supplementary material for: Prognosis prediction for end-stage pancreatic cancer with home medical care: A retrospective cohort study conducted at multiple institutions in Hiroshima
Source: BMC Palliat Care. 2026 May 11;25:194. doi: 10.1186/s12904-026-02015-1 (PMC13330228; doi:10.1186/s12904-026-02015-1)
Supplement: Supplementary file 1 — Additional file 1: Supplementary Table S1. Sensitivity analyses comparing model specifications. Supplementary Table S2. Comparison with patients aged ≥ 75 years. [file 12904_2026_2015_MOESM1_ESM.docx]

Supplementary Table S1. Sensitivity analyses comparing model specifications

| Model | Key predictors included | AIC | Variable | HR | 95% CI | p value |
| --- | --- | --- | --- | --- | --- | --- |
| Primary model (median-based) | Prior opioid use, peritoneal dissemination, mGPS of 2, NLR > median, others as covariates | 1340.9 | **Use of opioids before home care** | **1.44** | **1.00–2.07** | **0.047** |
|  |  |  | **Peritoneal dissemination** | **1.50** | **1.07–2.11** | **0.020** |
|  |  |  | **mGPS of 2** | **2.16** | **1.43–3.28** | **<0.001** |
|  |  |  | NLR (>median) | 1.33 | 0.93–1.91 | 0.118 |
| Sensitivity model A (NLR > 5) | Same covariates but NLR dichotomized at 5 | 1342.5 | Use of opioids before home care | 1.430 | 0.99–2.05 | 0.055 |
|  |  |  | **Peritoneal dissemination** | **1.479** | **1.05-2.09** | **0.026** |
|  |  |  | **mGPS of 2** | **2.14** | **1.41–3.24** | **0.001** |
|  |  |  | NLR (> 5) | 1.37 | 0.95–1.97 | 0.092 |
| Sensitivity model B (NLR continuous) | Same covariates but NLR modelled as continuous (per log2) | 1332.6 | Use of opioids before home care | 1.29 | 0.97–1.99 | 0.075 |
|  |  |  | Peritoneal dissemination | 1.36 | 0.96–1.91 | 0.083 |
|  |  |  | **mGPS of 2** | **1.89** | **1.24–2.88** | **0.003** |
|  |  |  | **NLR (continuous: per log2)** | **1.22** | **3.11–47.1** | **< 0.001** |

Abbreviations: AIC, Akaike Information Criterion; CI, confidence Interval; HR, hazard ratio; NLR, neutrophil-to-lymphocyte ratio; mGPS, modified Glasgow Prognostic Score

Bold text indicates statistical significance.

Supplement Table S2. Comparison with patients aged ≥ 75 years

| Variable | **>75 y** | **≤ 75 y** | p value |
| --- | --- | --- | --- |
| **Hypertension** | **39 (47.6%)** | **16 (18.6%)** | **< 0.001** |
| Diabetes myelitis | 28 (34.2%) | 31 (36.11%) | 0.872 |
| **Dementia** | **14 (17.1%)** | **3 (3.5%)** | **0.004** |
| Oral intake, severe | 28 (34.2%) | 30 (34.9%) | 1.000 |
| **Cancer-related pain** | **66 (80.5%)** | **79 (91.9%)** | **0.043** |
| Use of opioids | 69 (84.2%) | 77 (89.5%) | 0.897 |
| **Use of opioids before home medical care** | **32 (39.0%)** | **51 (59.3%)** | **0.009** |
| **Distal metastasis** | **57 (70.4%)** | **75 (89.3%)** | **0.003** |
| Liver metastasis | 41 (50.0%) | 56 (65.1%) | 0.061 |
| Peritoneal dissemination | 23 (28.1%) | 34 (39.5%) | 0.1427 |
| **mGPS 2** | **52 (63.4%)** | **69 (80.2%)** | **0.017** |
| **NLR > 5.1** | **33 (40.2%)** | **51 (59.3%)** | **0.020** |

Abbreviations: NLR, neutrophil-to-lymphocyte ratio; mGPS, modified Glasgow Prognostic Score

Bold text indicates statistical significance.
